# Complete closure of large rectal wound and perforation using a novel through-the-scope twin clip

**DOI:** 10.1055/a-2418-3116

**Published:** 2024-10-08

**Authors:** Ding Peng, Yinglin Niu, Ziyu Liu, Linchao Li, Rui Wang, Guangwei Qiu, Huihong Zhai

**Affiliations:** 1 71044 Gastroenterology, Xuanwu Hospital Capital Medical University, Beijing, China; 2 Gastroenterology, Beijing Friendship Hospital, Capital Medical University, Beijing, China

Endoscopic submucosal dissection (ESD) is an advanced endoscopic technique used to resect large or early cancerous rectal lesions. However, severe fibrosis or large size of lesion significantly increases the difficulty and risk of the procedure. In particular, postoperative perforation and closure of large wounds remain major clinical challenges. For small perforations, endoscopic closure with through-the-scope (TTS) and over-the-scope (OTS) clips is commonly used 1 . However, larger wounds or complex perforations may require specialized suturing devices or surgical repair. Previous reports have documented the use of the TTS twin clip (TTS-TC; Micro-Tech, Nanjing, China) for closing gastric or duodenal wounds 2 . This report presents a case of successful closure using TTS-TCs of a large rectal wound with perforation. This technique not only offers ease of operation but also excels in closing large wound areas, providing a new therapeutic option, and our report offers practical evidence for similar cases.

A 72-year-old man presented with a history of altered bowel habits for 2 months. Colonoscopy performed at a local hospital had revealed a large irregular mass 10 cm from the anus, and pathology findings indicated a villous tubular adenoma with high grade intraepithelial neoplasia. The patient was referred to our hospital for endoscopic treatment.

Preoperative contrast-enhanced abdominal computed tomography (CT) showed no enlarged lymph nodes. Colonoscopy revealed a large Is-type elevated lesion in the rectum, approximately 4 × 5 cm in size, with a mucosal white plaque at the base ( Fig. 1 ). After submucosal injection of sodium hyaluronate solution, a positive lifting sign was observed. ESD was performed using a combination of tissue clips and a rubber band for auxiliary traction. Significant central fibrosis of the lesion was noted during the procedure. The lesion was successfully resected, with a wound size of approximately 5 cm, extending to 3/4 of the rectal circumference. A 1-cm perforation was observed in the center of the wound, with the serosal layer exposed ( Fig. 2 ).

**Fig. 1:**
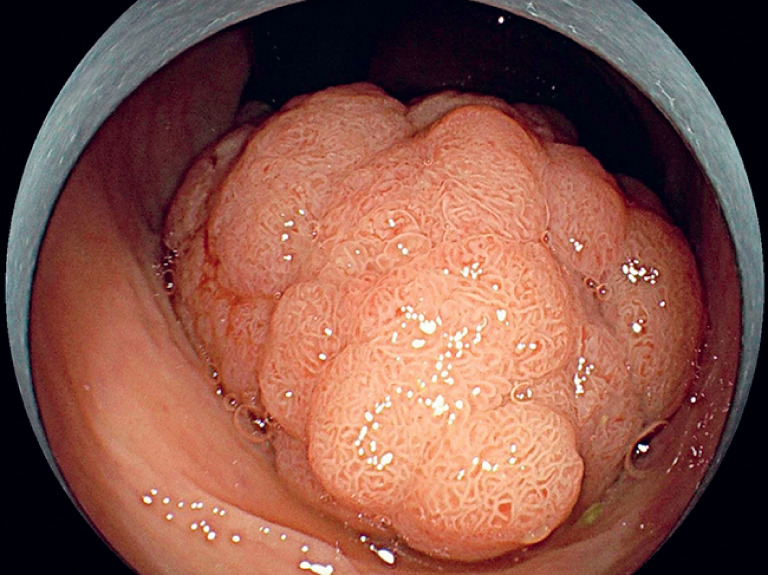
A rectal lesion 10 cm from the anus in a 72-year-old man.

**Fig. 2:**
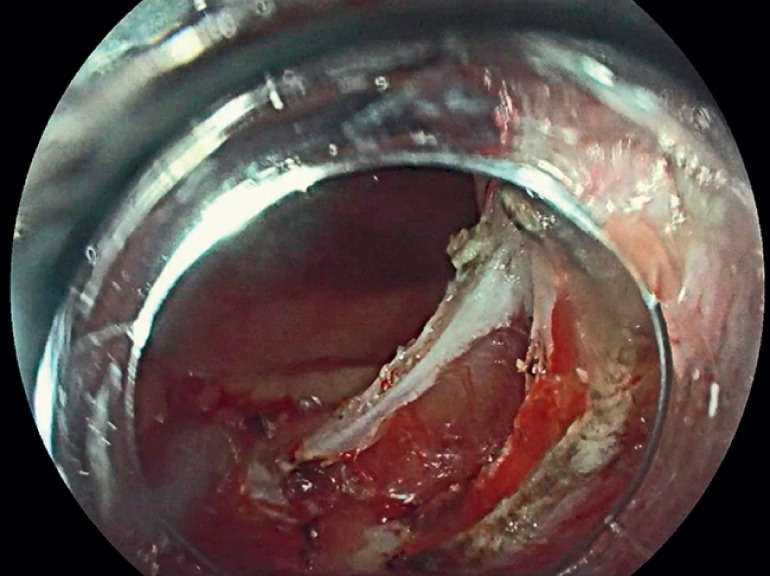
Post endoscopic submucosal dissection (ESD) wound extending to 3/4 of the rectal circumference with perforation at the center exposing the serosa.

A TTS-TC was inserted through the endoscopic biopsy channel to close the perforation. First, the entire perforation edge and adjacent normal mucosa were accurately clipped, positioning the clip at the anal side of the lesion, then the TTS-TC was released ( Fig. 3 , Video 1 ). Considering the large wound size, two additional TTS-TCs were used to clip the perforation site and adjacent normal mucosa, reducing the wound size to one manageable by using conventional TTS clips. Finally, conventional TTS clips were used to completely close the wound ( Fig. 4 ).

**Fig. 3:**
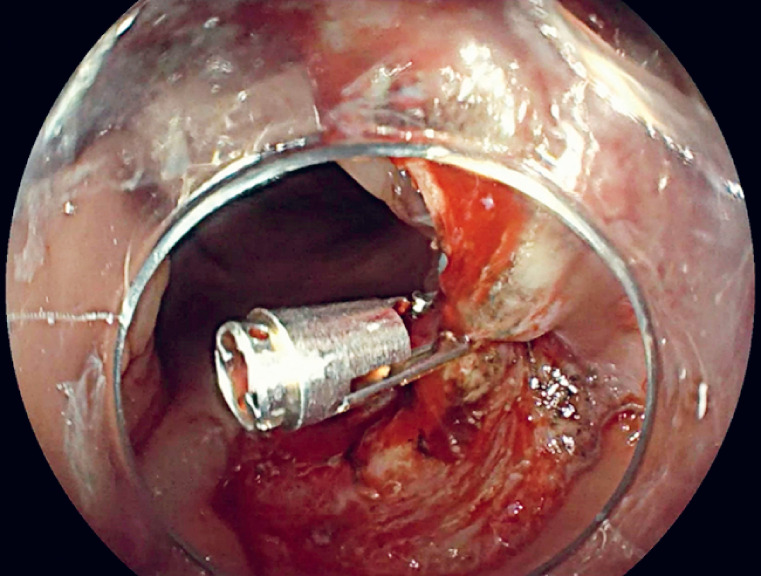
Perforation closed and partial wound closure.

**Fig. 4:**
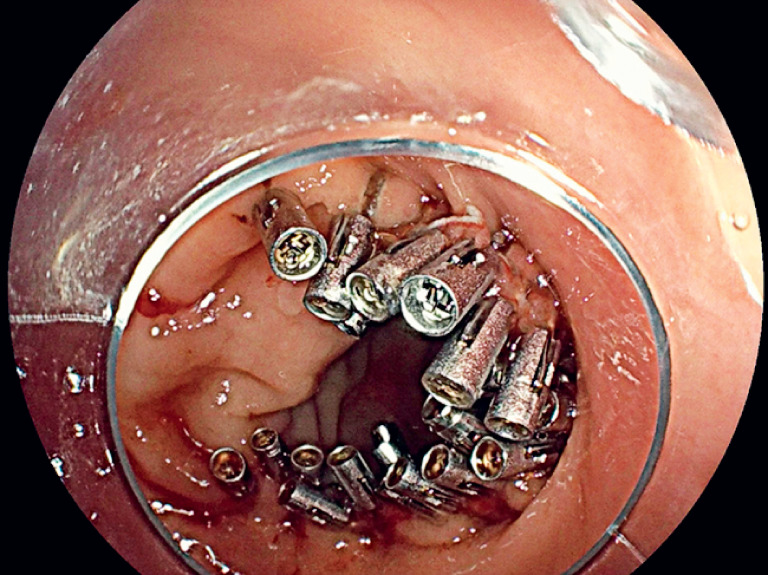
Complete sealing of wound and perforation.

Complete sealing of large rectal wound and perforation using through-the-scope twin clips (TTS-TCs).Video 1

The patient was kept fasting for 4 days postoperatively. On the second postoperative day, the white blood cell count was slightly elevated, peaking at 10.92 × 10 ^9^ /L, without fever, chills, or other discomfort. Abdominal CT on postoperative day 7 showed no significant abnormalities, and the white blood cell count returned to normal. The patient was discharged on postoperative day 8. The postoperative pathological results showed a traditional serrated adenoma with negative margins.

The TTS-TC is a precise, efficient, and safe device for endoscopic closure of large rectal wounds with perforation.

Endoscopy_UCTN_Code_TTT_1AQ_2AK

## Notes

Endoscopy E-Videos https://eref.thieme.de/e-videos
*E-Videos* is an open access online section of the journal *Endoscopy* , reporting on interesting cases and new techniques in gastroenterological endoscopy. All papers include a high-quality video and are published with a Creative Commons CC-BY license. Endoscopy E-Videos qualify for HINARI discounts and waivers and eligibility is automatically checked during the submission process. We grant 100% waivers to articles whose corresponding authors are based in Group A countries and 50% waivers to those who are based in Group B countries as classified by Research4Life (see: https://www.research4life.org/access/eligibility/ ). This section has its own submission website at https://mc.manuscriptcentral.com/e-videos .
